# The impact of prenatal amoxicillin exposure at different doses, stages, and courses on offspring ovarian development

**DOI:** 10.1186/s10020-025-01322-2

**Published:** 2025-07-22

**Authors:** Jing Huang, Yating Li, Ming Zhang, Tiancheng Wu, Yuanzhen Zhang, Hui Wang

**Affiliations:** 1 Department of Obstetrics and Gynaecology, Zhongnan Hospital of Wuhan University, Wuhan, 430071 China; 2 Department of Otorhinolaryngology Head and Neck Surgery, Zhongnan Hospital of Wuhan University, Wuhan, 430071 China; 3 Department of Pharmacology, Basic Medical School of Wuhan University, Wuhan, 430071 China; 4 Hubei Provincial Key Laboratory of Developmentally Originated Disease, Wuhan, 430071 China; 5 Department of Pharmacology, School of Basic Medical Sciences, Wuhan University, Wuhan, 430071 China

**Keywords:** Prenatal amoxicillin exposure, Ovarian developmental toxicity, Pregranulosa cells, Oocytes, Medication safety

## Abstract

**Background:**

Amoxicillin, a commonly used broad-spectrum penicillin antibiotic in pregnancy, has sparked controversy regarding its impact on fetal growth and development. There remains a lack of systematic research on the specific influence of prenatal amoxicillin exposure (PAmE) on the ovarian development of the offspring, as well as the precise " toxicity windows “.

**Methods:**

We established PAmE mouse models at different stages [(gestational day, GD) 10–12, GD13-15 or GD16-18], doses (75, 150 or 300 mg/kg·d), and courses (single/multiple courses). On GD18, fetal serum and ovaries were collected to assess changes in serum estradiol levels and evaluate ovarian morphology, pregranulosa cell function, and oocyte-related parameters.

**Results:**

PAmE led to pathological damage in fetal mouse ovaries, characterized by disrupted germ cell cysts and reduced the number of germ cells. Cell proliferation was enhanced while apoptosis was reduced. Moreover, PAmE upregulated the expression of pregranulosa cell steroid synthesis-related genes (e.g., *Sf1*,* Star*,* P450scc*) in the fetal ovaries, particularly in the high-dose groups at all gestational stages. The expression of the oocyte marker gene Figlα increased in all PAmE groups, while follicle development-related genes (*Nobox* and *Bmp15*) were downregulated, particularly during early to mid-pregnancy and in the single-course exposure groups. Further investigation revealed that PAmE enhanced IGF1 expression in fetal ovaries and inhibited the Pten-Akt-Foxo3a signaling pathway.

**Conclusions:**

Amoxicillin exhibits ovarian developmental toxicity, influencing fetal ovarian cell proliferation, apoptosis, pregranulosa cell estrogen synthesis, oocyte numbers, and follicle assembly. This study provides evidence guiding the rational use of amoxicillin in pregnancy and assessing potential ovarian development risks.

**Graphical Abstract:**

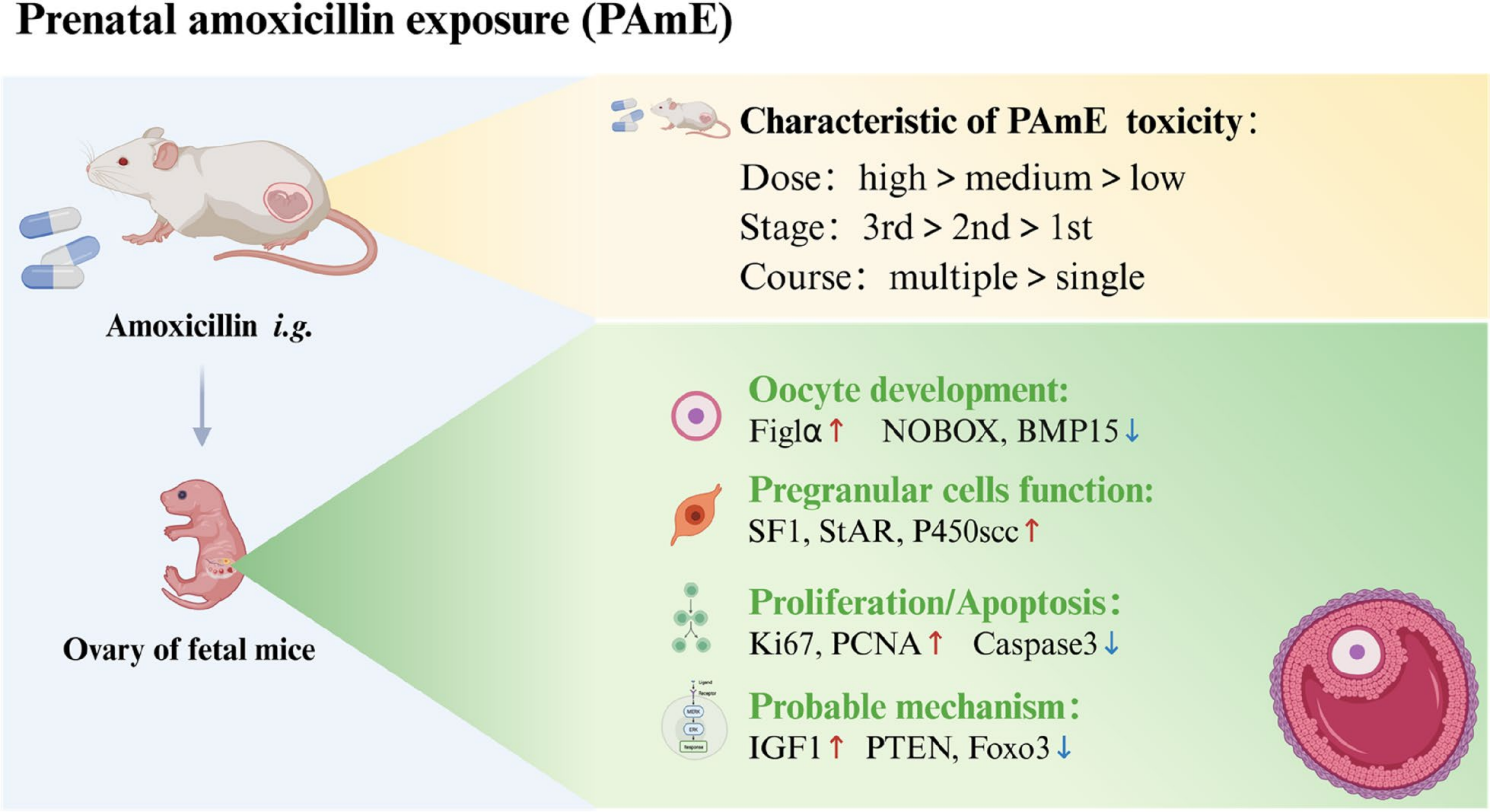

## Introduction

Pregnancy is a crucial time for fetal development, but it’s vulnerable to external influences (Wang et al. 2019). The “Developmental Origins of Health and Disease” (DOHaD) holds that these adverse factors endanger the growth and development of the fetus function in an “intrauterine programming” manner. It has a profound impact on the health outcome after birth and even the health throughout the entire life period and affects the development and function of multiple organs in the offspring (Lejonklou et al. 2016; Lv et al. 2021; Zhang et al. 2016), including the development of the female reproductive system (Lv et al. 2021). The ovary plays a vital role in female reproduction by producing oocytes, hormones, and maintaining reproductive health. The increasing prevalence of ovarian diseases like premature ovarian insufficiency poses significant risks to women’s reproductive and long-term health. Research shows that exposure to toxins, drugs, and unhealthy habits during pregnancy can affect the offspring’s ovarian structure, development, and reproductive health in adulthood (Aiken et al. 2015; Camlin et al. 2017). Understanding these impacts is crucial for preserving reproductive health and overall well-being.

Though pregnant individuals are generally careful with medications, antibiotics are often necessary for treating infections, reducing postpartum infections, and preventing neonatal infections (Mueller et al. 2017). Among the top 20 drugs prescribed during pregnancy, 8 are antibiotics, with amoxicillin being the most used, at a rate of 18.0% (Ferretti et al. 2018). Amoxicillin is a small-molecule penicillin (Ben et al. 2020), it swiftly passes through the placenta into the fetal body (Nau 1987; Pacifici and Nottoli 1995). However, research on its effects during pregnancy yields mixed results. Some studies suggest that prenatal amoxicillin exposure (PAmE) may impact pregnancy outcomes, leading to preterm birth, miscarriage, stillbirth, and developmental abnormalities (Duman et al. 2022; Lin et al. 2012; Metsala et al. 2015). However, clinical analyses of medical records have not consistently found an increased risk of congenital disabilities or low birth weight with PAmE (Chen et al. 2019; Cooper et al. 2009; Boeckel et al. 2014), sparking ongoing debate about its safety during pregnancy. Previous animal studies have found that azithromycin during pregnancy has ovarian developmental toxicity (Li et al. 2024), but whether PAmE affects the ovarian development of the offspring has not been reported yet.

During pregnancy, amoxicillin can be taken as a single 3 g dose or as 750 mg tablets three times daily for four days (Gerstner et al. 1989). Pregnant women are susceptible to listeriosis, a disease often unnoticed until later pregnancy stages (Wang et al. 2021). To prevent fetal infection, early antibiotic treatment like amoxicillin is suggested (Madjunkov et al. 2017). However, the impact and risks of drugs often hinge on dosage, higher doses can pose more risks to the fetus (Zhang et al. 2017). This study aims to use different doses, stages, and treatment courses of amoxicillin in mouse models to understand its effects on fetal ovarian development. This research will help in recommending safe medication during pregnancy and assessing the risk of fetal ovarian toxicity.

## Materials and methods

### Chemicals

Amoxicillin was provided by Hainan General Sanyo Pharmaceutical Co., Ltd (Hainan, China). The mouse estradiol enzyme-linked immunoassay kit (Item No. AR E-8800) was supplied by Beijing North Institute of Biological Technology Co., Ltd. (Beijing, China). Isoflurane (Item No. R510-22) was provided by RWD Life Science Co., Ltd. (Shenzhen, China). TRIzol TM reagent (Item No. 15596026) was supplied by Thermo Fisher Scientific Co., Ltd. (Waltham, MA, USA). HiScript III RT SuperMix for qPCR (Item No. R323-01) and Cham QTM Universal SYBR^®^ qPCR Master Mix (Item No. Q712-02) were provided by Vazyme Biotech Co., Ltd. (Nanjing, China). All reverse transcription real-time quantitative polymerase-chain-reaction (RT-qPCR) primers were synthesized by TIANYIHUIYUAN Gene Biotechnology Co., Ltd. (Wuhan, China). Antibodies for PCNA (No. A0264), STAR (No. A16432), Caspase3 (No. A2156), and MVH (No. A15624) were purchased from ABclonal Technology Co., Ltd. (Wuhan, China). All other chemicals and materials were of analytical grade.

### Animals and treatment

**Fig. 1:**
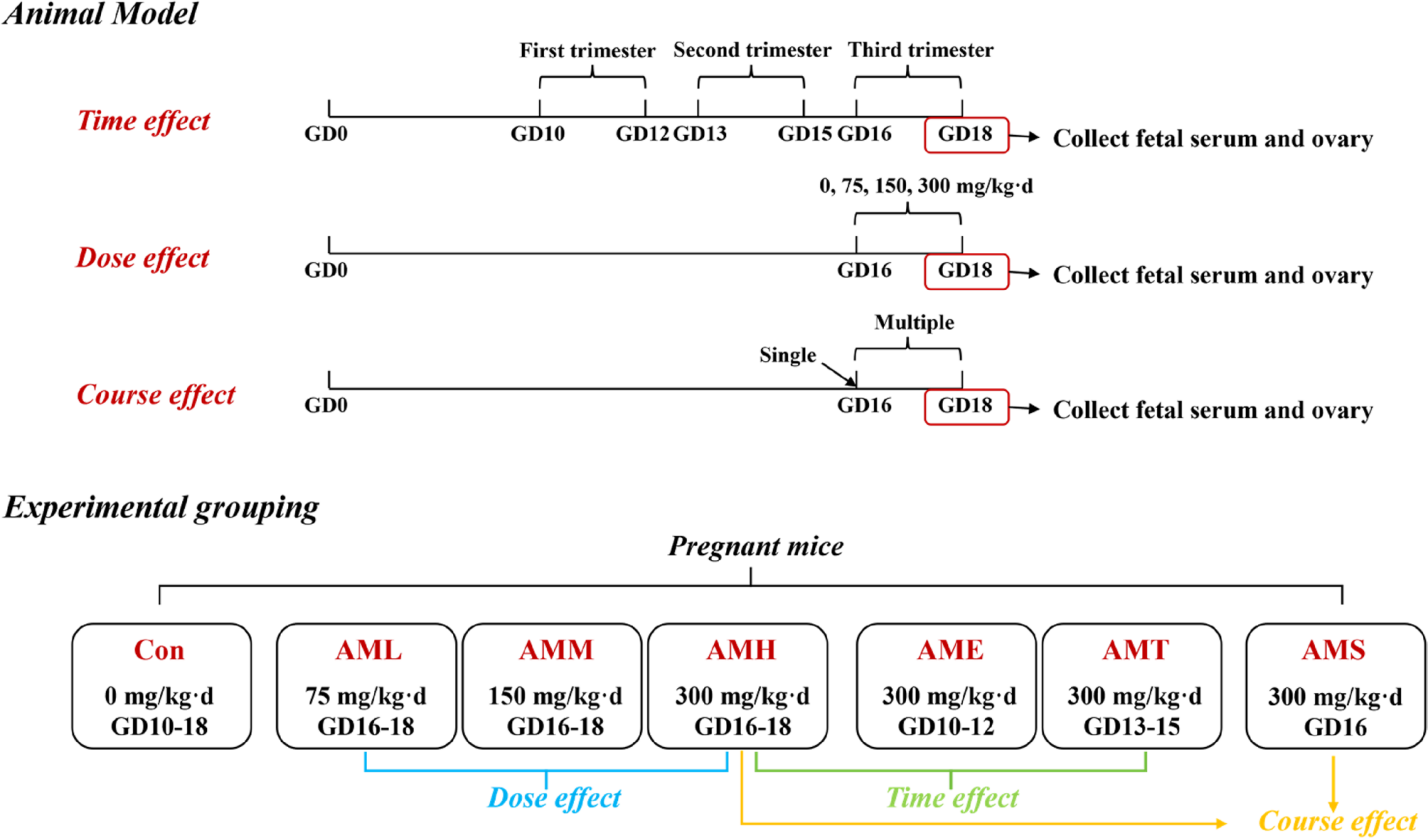
The schematic illustration of animal treatment and experiment grouping. GD, gestational day; CON, the control group was administered the same volume of sodium carboxymethylcellulose; AML, amoxicillin low-dose exposure; AMM, amoxicillin medium-dose exposure; AMH, amoxicillin high-dose exposure; AME, amoxicillin early exposure; AMT, amoxicillin terminal exposure; AMS, amoxicillin single exposure

This study was conducted with approval from the Ethics Committee for Experimental Animal Welfare of Zhongnan Hospital of Wuhan University and followed strict guidelines from the National Institutes of Health and ARRIVE. The experiments were carried out at the Animal Experimental Center of Wuhan University, which is accredited by AAALAC International. Eight-week-old SPF Kunming mice (2020-0018, Certification No. 42000600042125, License No. SCXK (Hubei) were obtained from Beijing Sibeifu Biotechnology Co., Ltd. After acclimatization, they were paired for mating, and gestational day (GD) 0 was marked by observing vaginal plugs. Pregnant mice were transferred to individual cages and randomly divided into six groups (*n* = 12 per group) to receive amoxicillin at different stages (GD10-12, GD13-15, or GD16-18), doses (75, 150, or 300 mg/kg·d) at GD16-18, or courses (GD16 or GD16-18) at 300 mg/kg·d. The dose of amoxicillin used in the mouse experiments of this study was determined based on the most used treatment regimen in humans. The human-to-animal dose conversion formula was applied: $D_{\text{h}}\;=\;D_{\text{a}}\times\frac{\mathrm{Animal}\;K\mathrm m}{\mathrm{Human}\;K\mathrm m}$, where *D*_h_ (mg/kg) represents the human dose, *D*_a_ (mg/kg) represents the animal dose, and the correction factor (*K*_m_) is the ratio of body weight to surface area (Flint and Hall 2022). Clinically, the standard oral amoxicillin dosage for pregnant women is 500 mg every 8 h (equivalent to 1500 mg/day or approximately 25 mg/kg·d for a 60 kg woman) (https://www.drugs.com/dosage/amoxicillin.html). Using the dose conversion formula, with *K*_m_ values of 37 for humans and 3 for mice, the equivalent daily exposure of 25 mg/kg amoxicillin in humans corresponds to approximately 308.3 mg/kg in mice $D_{\text{h}}\;=\;D_{\text{a}}\times\frac{\mathrm{Animal}\;K\mathrm m}{\mathrm{Human}\;K\mathrm m}\;=25\left(\mathrm{mg}/\mathrm{kg}\right)\;\times\frac{37}3\approx\;308.3\;\mathrm{mg}/\mathrm{kg}$. Therefore, the administration dose for mice in this study was set at 300 mg/kg·d, which was used to evaluate its therapeutic course and time-dependent effects. Additionally, high, medium, and low doses were established at 300, 150, and 75 mg/kg·d, respectively, to further investigate the dose-response relationship of amoxicillin, and explore the clinical safe range. The control group received sodium carboxymethylcellulose (10 ml/kg·d) from GD10 to GD18. On GD18, pregnant mice were euthanized with 3% isoflurane anesthesia, and viable fetus counts were recorded. The right ovary from each litter was used for morphological analysis, while the remaining ovaries were frozen in liquid nitrogen and stored at −80°C. Blood samples were collected for serum extraction (Fig. 1).

### Hormonal level measurements

According to the manufacturer’s instructions, enzyme-linked immunosorbent assay (ELISA) kit was used to determine the concentration of estradiol in the collected serum from fetal and pregnant mice.

### Hematoxylin-eosin (HE) staining

The fetal ovaries were fixed overnight in a solution of 4% paraformaldehyde. Standard HE staining was then performed on the fixed ovaries. Each group’s ovaries were sectioned into slices using a microtome to obtain maximum cross-sectional views. Observation and photography were done using an Olympus AH-2 optical microscope. Another experimenter conducted blind examinations. Microphotography was performed for each slide (*n* = 5), and parameters like maximum cross-sectional area and diameter were assessed. To count and classify oocytes, five units per square were randomly chosen from the interstitial tissue area of each slide. Image J analysis was used to calculate the number of oocytes per unit square in five different slices, with each section measuring 4 × 10^4^ µm^2^.

### Immunofluorescence staining

Ovarian tissue was embedded in paraffin, sectioned at 5 μm, deparaffinized in xylene and rehydrated. Samples were thermal remediated in citrate buffer (10 mM, pH 6.0) at 65 °C for 5 h and then rinsed three times with PBS. Sections were incubated with primary antibodies (1:100): anti-MVH, anti-StAR, or anti-PCNA overnight at 4 °C after blocking with 5% BSA for 30 min at room temperature. Subsequently, sections were then incubated with fluorescent secondary antibody (1:200) for 2 h at room temperature. The sections were then stained with 4’,6-diamidino-2-phenylindole (DAPI) (1 µg/ml) for 10 min. All images were taken with a Light Microscope Olympus AH-2 (Olympus, Tokyo, Japan). The Olympus software was used for the analysis of stained images.

### Total RNA extraction, reverse transcription, and RT-qPCR for ovary

Each sample, representing the ovary tissue of female fetal mice from each litter, was homogenized after the addition of TRIzol TM reagent and zirconium grinding beads. Total RNA was extracted following standard procedures with chloroform and isopropanol. RNA concentration and purity were assessed using a NanoDrop 2000 spectrophotometer, adjusted to 1 mg/ml, and converted to cDNA. Primers (listed in Table 1) were designed and verified using Primer Premier 5.0 (Premier Biosoft International, CA) and NCBI BLAST database, respectively. RT-qPCR was conducted with ChamQ Universal SYBR qPCR Master Mix on a QuantStudio^®^5 Real-Time PCR System (Thermo Fisher Scientific, Waltham, MA, USA). glyceraldehyde-3-phosphate dehydrogenase (GAPDH) was used as a reference gene for normalization, employing the 2-ΔΔCt method for quantification.

**Table 1:** Mouse oligonucleotide primers in RT-qPCR

Genes	Forward primer	Reverse primer
*Gapdh*	GCAAGTTCAATGGCACAG	GCCAGTAGACTCCACGACA
*Pcna*	CACTCCACTGTCTCCTACA	GCCTAAGATGCTTCCTCATC
*Ki67*	CTGGTCTCAAAGGACCAATC	CTCTTCATCTGCCTCTACTTTC
*Caspase3*	GATATTGTTAGCGGTTCCTGTG	AACCAGGCTGTCGTCTAA
*Sf1*	CCAGTACGGCAAGGAAGA	GAGGCTGAAGAGGATGAGGA
*Star*	GGGAGATGCCTGAGCAAAGC	GCTGGCGAACTCTATCTGGGT
*P450scc*	GCTGCCTGGGATGTGATTTTC	GATGTTGGCCTGGATGTTCTTG
*Cyp19*	ATGGGCCTCCTTCTCCTGAT	CAGGCACTTCCAATCCCCAT
*Nobox*	GACATGGGACCTCAGGATTA	GAGTCTTCTGGTGGTAGAAATG
*Figlα*	AGAGCGTGAGCGGATAAA	CCAGAACACAGCCAAGTATC
*Bmp15*	GTGCTCAGGCTAAACTTCTT	GGAGGGAACACTGGTTATTT
*Igf1*	TGCAGGCAGCCTAAGCACCTACCTC	CCCAGGGTCCATTTTCCAACCTT
*Pten*	CAGTAGAGGAGCCATCAAATC	GAGTCAGTGGTGTCAGAATATC
*Foxo3*	GAGTGACTCCAGCCTTG	ATTCCAAGCTCCCATTGAAC

*Gapdh* Glyceraldehyde phosphate dehydrogenase, *Pcna* proliferating cell nuclear antigen, *Sf1* steroidogenic factor 1, *Star* Steroidogenic acute regulatory protein, *P450scc* cytochrome P450 cholesterol side chain cleavage, *Cyp19 *cytochrome P450 family 19, *Nobox* *Nobox* oogenesis homeobox, *Figlα* Factor in the germline alpha, *Bmp15* Bone morphogenetic protein 15, *Igf1 *Insulin-like growth factor 1, *Pten* Phosphatase and tensin homolog, *Foxo3 *FORKHEAD box protein O3

### Statistical analysis

Data analysis was performed using Excel (Microsoft, Redmond, WA, USA), Prism (GraphPad Software, La Jolla, CA, USA), and Image J. Quantitative data are presented as mean ± S.E.M. The comparison of means between two groups was conducted using a two-tailed Student’s T-test. A *P*-value of less than 0.05 was considered statistically significant.

## Results

### Effects of different doses, stages, and courses of PAmE on fetal ovarian morphological development

**Fig. 2:**
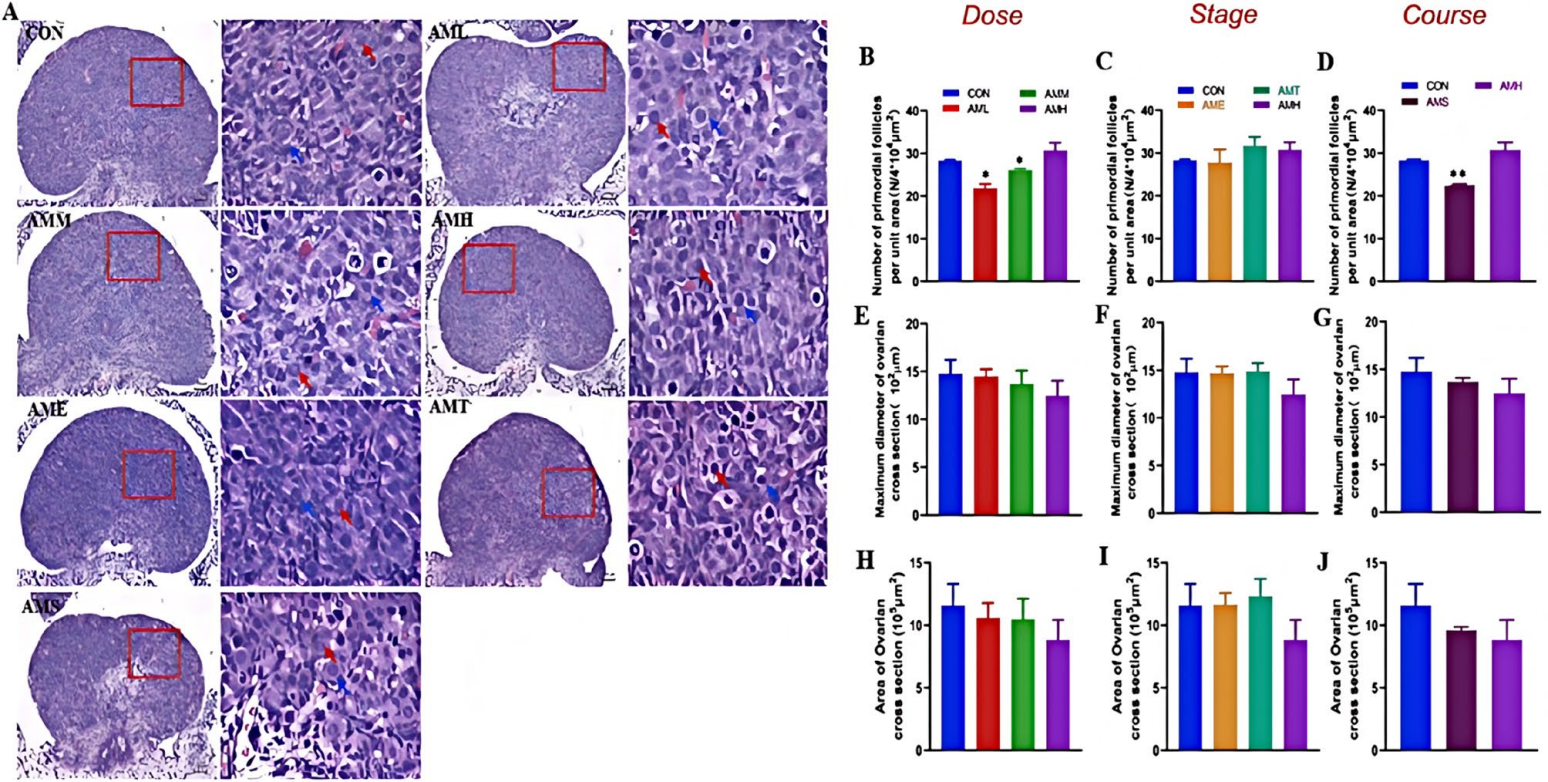
Effects of different doses, stages and courses of prenatal amoxicillin exposure on the fetal ovarian morphology. **A** Ovarian morphology (HE, 200×, 400×, *n =* 5, red arrow showed the oocytes, blue arrow showed the ovarian pre-granulosa cells); (**B**-**D**) Number of primordial follicles per unit area (N/10^4^ µm^2^); (**E**-**G**) Maximum diameter of ovarian cross section (µm); (**H**-**J**) Area of ovarian cross Sect. (10^5^ µm^2^). Mean ± S.E.M., *n* = 5. ^*^P < 0.05, ^**^P < 0.01 vs. CON

Since the maximum diameter, cross-sectional area and the number of oocytes of the fetal ovaries are the important indicators for evaluating the growth and development of the fetal ovaries, we observed the changes in the morphological development of the fetal ovaries based on the above PAmE models with different doses, stages and courses. We found that compared to the control group, PAmE significantly reduced the number of oocytes per unit area, particularly in the low and mid-dose groups (*P* < 0.05, Fig. 2B). However, it did not significantly affect oocyte quantity across different exposure stages (Fig. 2C). A single course of PAmE caused a significant decrease in oocyte number (*P* < 0.01, Fig. 2D), while multiple courses had no significant effect. The ovarian size and area showed no significant differences between the PAmE and control groups (Fig. 2E-J). HE stains revealed disrupted germ cell cyst arrangement, leading to decreased oocyte numbers and premature primordial follicle assembly. These findings suggest that PAmE can impair fetal ovarian morphology, especially in late-pregnancy, low to mid-dose, and single-course groups.

### Effects of different doses, stages, and courses of PAmE on fetal ovarian cell proliferation and apoptosis

**Fig. 3:**
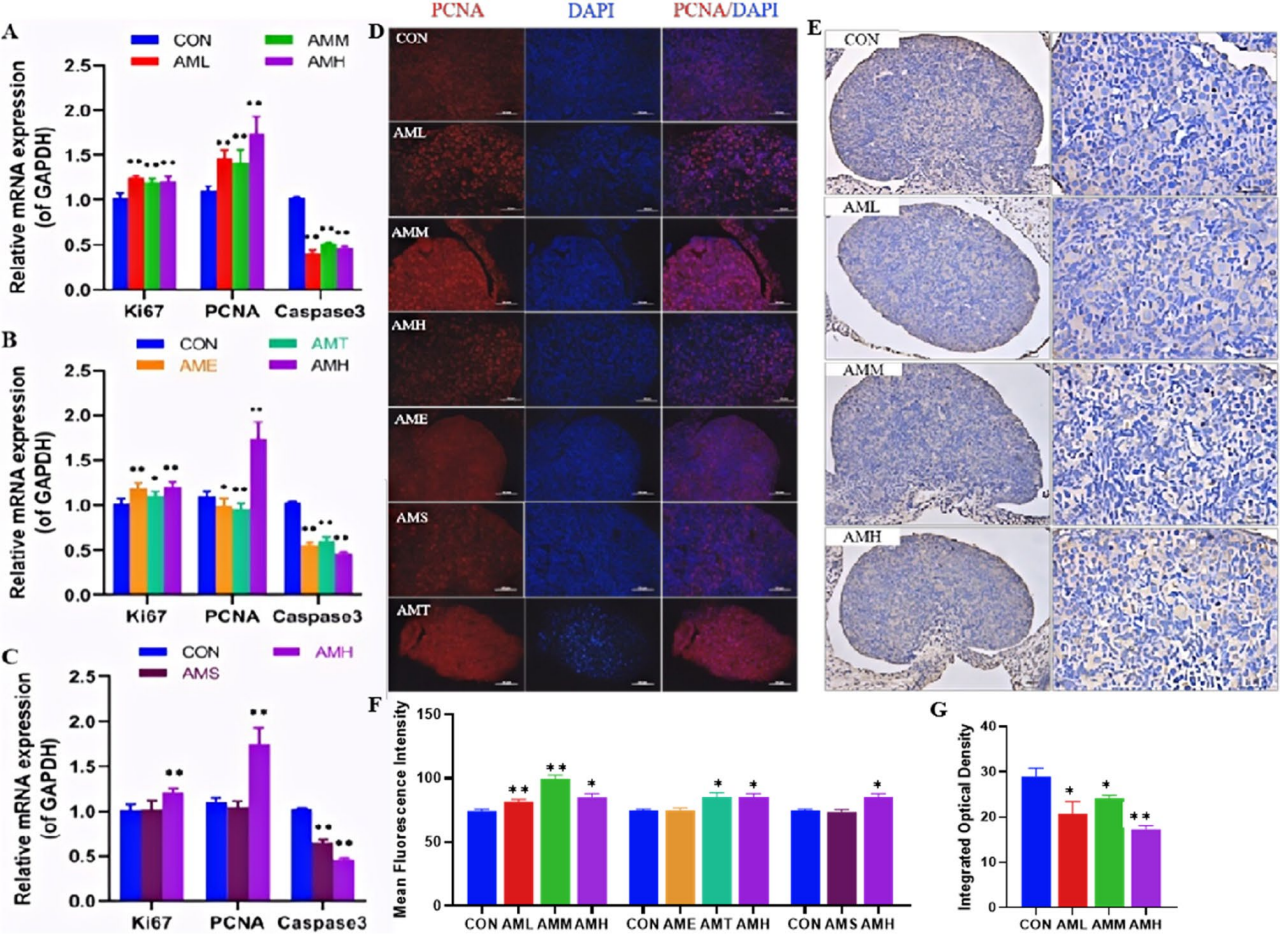
Effects of different doses, stages and courses of prenatal amoxicillin exposure on proliferation and apoptosis in fetal ovary.**A**-**C** Relative mRNA level of Ki67, PCNA, and Caspase3, *n*=12 per group; (**D**, **E**) Representative immunofluorescence images and mean fluorescence intensity of PCNA, n=5; (**E**, **G**) Immunohistochemistry and quantification of Caspase3 in fetal ovary, *n*=5. Mean ± S.E.M., ^*^P<0.05, ^**^P<0.01 *vs. *CON

To determine whether PAmE has an impact on ovarian cell proliferation and apoptosis, we assessed the mRNA expressions of *Ki67*,* Caspase3*, and *Pcna* in fetal ovaries. We found that compared to the control group, the mRNA expressions of *Ki67* and *Pcna* were increased at low, medium, and high-dose groups (*P* < 0.01, Fig. 3A), while the mRNA expression of *Caspase3* was decreased (*P* < 0.01, Fig. 3A). Comparing the different pregnancy stages, we found that mRNA expression of the *Ki67* was increased (*P* < 0.01, *P* < 0.05, Fig. 3B) and the *Caspase3* was decreased (*P* < 0.01, Fig. 3B) in each group compared with the control group. Interestingly, *Pcna* expressions decreased in early and mid-pregnancy but increased in late-pregnancy (*P* < 0.01, Fig. 3B). Multiple PAmE courses (AMH) increased *Ki67* and *Pcna* while decreasing the Caspase3 mRNA levels (*P* < 0.01, Fig. 3C). IF and IHC staining also showed changes in the protein expression of PCNA and CASPASE3 (Fig. 3D, E). In summary, all dose levels, multi-course treatments, and gestational exposure to PAmE promoted Ki67 expression while inhibiting Caspase3 expression. Notably, in the high-dose, multi-course PAmE group, PCNA expression decreased during the first and second trimesters but increased during the third trimester of exposure.

### Effects of different doses, stages, and courses of PAmE on fetal ovarian steroidogenesis

**Fig. 4:**
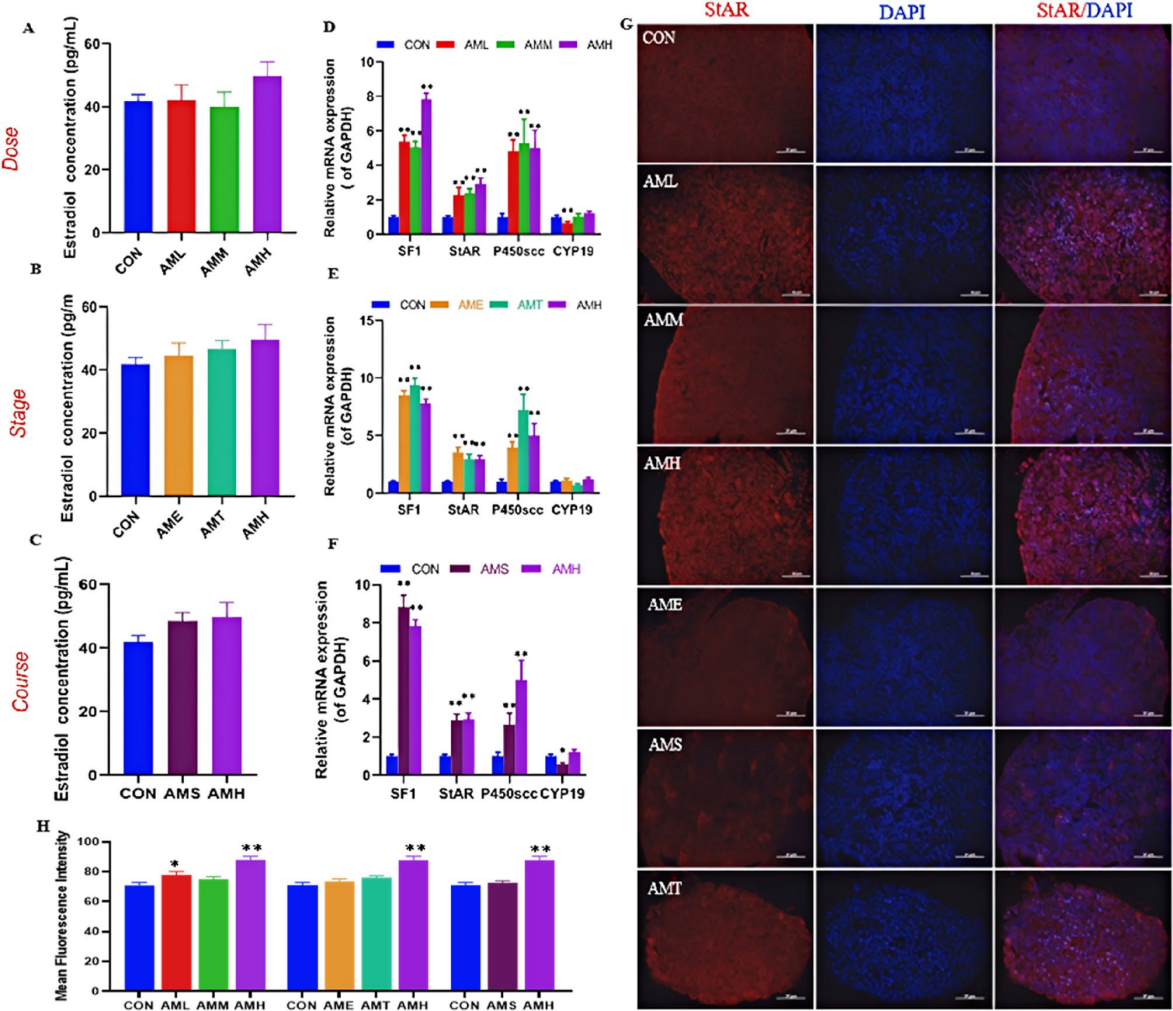
Effects of different doses, stages and courses of prenatal amoxicillin exposure on estradiol synthesis function in fetal ovary. **A**-**C** Estradiol concentration of fetal serum, *n* = 8; (**D**-**F**) Relative mRNA level of *SF1*, *P450scc*, *Star*, *CYP19* in different groups, *n* = 12 per group; (**G**, **H**) Representative immunofluorescence images and mean fluorescence intensity of StAR, *n* = 5. Mean ± S.E.M., ^*^P < 0.05, ^**^P < 0.01 *vs*. CON

Estrogen, vital hormones produced by ovarian granulosa cells, were studied regarding their response to PAmE in this research. While serum estradiol levels showed no significant change across PAmE and control groups, mRNA expression of ovarian steroidogenic enzymes like *Sf1*, *Star*, and *P450scc* increased in all dose groups (*P* < 0.01, Fig. 4D). Particularly, the low-dose group (AML) exhibited a significant decrease in *Cyp19* mRNA expression (*P* < 0.01, Fig. 4D). Throughout various pregnancy stages, PAmE led to a substantial rise in the mRNA expression of ovarian *Sf1*, *Star*, and *P450scc* (*P* < 0.01, Fig. 4E). Furthermore, high-dose (AMH) and single-course (AMS) treatments notably increased the mRNA expression of these enzymes (*P* < 0.01, Fig. 4F), with AMS also decreasing *Cyp19* mRNA expression significantly (*P* < 0.05, Fig. 4F). Immunofluorescence confirmed increased protein expression of STAR in AML and AMH groups (Fig. 4G). Overall, clinical doses of PAmE, as well as single or multiple courses, positively impacted fetal ovarian steroidogenic function, especially promoting SF1, StAR, and P450scc expression.

### Effects of different doses, stages, and courses of PAmE on fetal ovarian oocyte and follicle development

**Fig. 5:**
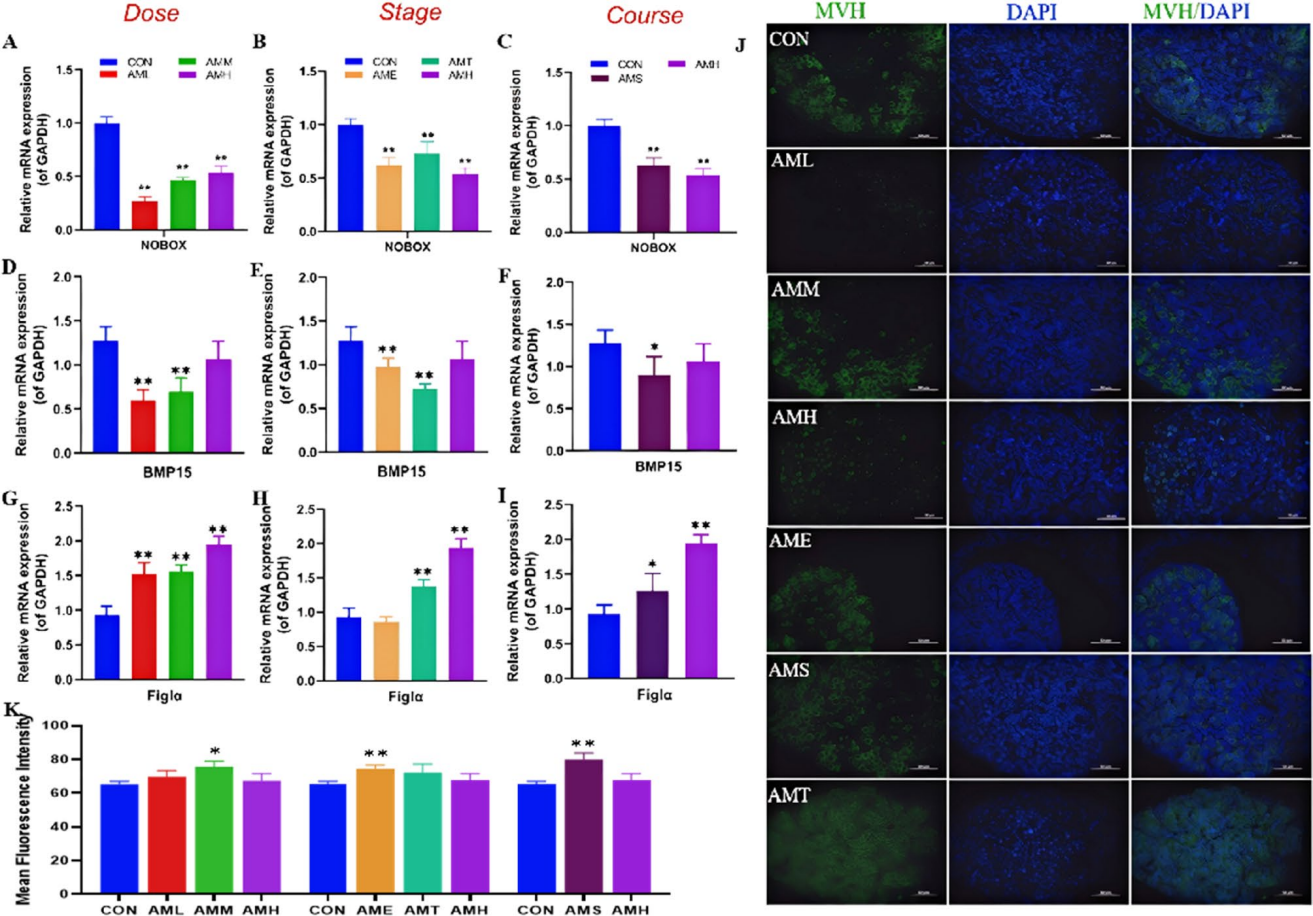
Effects of different doses, stages, and courses of prenatal amoxicillin exposure on oocyte function and follicle development in fetal ovary. **A**-**I** Relative mRNA level of *NOBOX*, *BMP15* and *Figlα* in fetal ovary, *n* = 12 per group; (**J**, **K**) Representative immunofluorescence images and mean fluorescence intensity of MVH (400×), *n* = 5. Mean ± S.E.M., ^*^P < 0.05, ^**^P < 0.01 *vs*. CON

Oocyte development is vital for ovarian reproductive function, with Figlα being a marker gene for oocyte development. We investigated how PAmE, at different doses, stages, and courses, affects *Figlα* mRNA expression. Higher PAmE doses led to increased *Figlα* mRNA expression (*P* < 0.05, Fig. 5G). Exposure during mid and late pregnancy also increased *Figlα* mRNA expression, especially in late pregnancy (AMH) (*P* < 0.01, Fig. 5B, H). Both single and multiple-course exposures increased *Figlα* mRNA expression (*P* < 0.05, *P* < 0.01, Fig. 5C, I). This suggests that PAmE promotes fetal ovarian oocyte development.

We also examined the effect of PAmE on *Bmp15* and *Nobox*, key factors associated with fetal follicle development. The low and mid-dose groups (AML and AMM) decreased *Bmp15* and *Nobox* mRNA expression (*P* < 0.01, Fig. 5F), while the high-dose group decreased *Nobox* without affecting *Bmp15* mRNA expression (Fig. 5F). Early and middle pregnancy exposure reduced *Bmp15* and *Nobox* mRNA expression, whereas late pregnancy exposure reduced *Nobox* mRNA expression (*P* < 0.01, Fig. 5E). Single-course exposure (AMS) reduced *Bmp15* and *Nobox* mRNA expression, and the effect on *Bmp15* was more pronounced in the multi-course group (*P* < 0.05, Fig. 5H). Immunofluorescence staining confirmed that mouse Vasa homologue (MVH) protein expression increased in the oocytes of AMM, AME, and AMS groups. In conclusion, PAmE may interfere with oocyte development and facilitate follicular development, especially at low doses, during the first to second trimester of pregnancy, or following a single-course exposure.

### Effects of different doses, stages, and courses of PAmE on fetal ovarian IGF1/PTEN-Akt-Foxo3 pathway

**Fig. 6:**
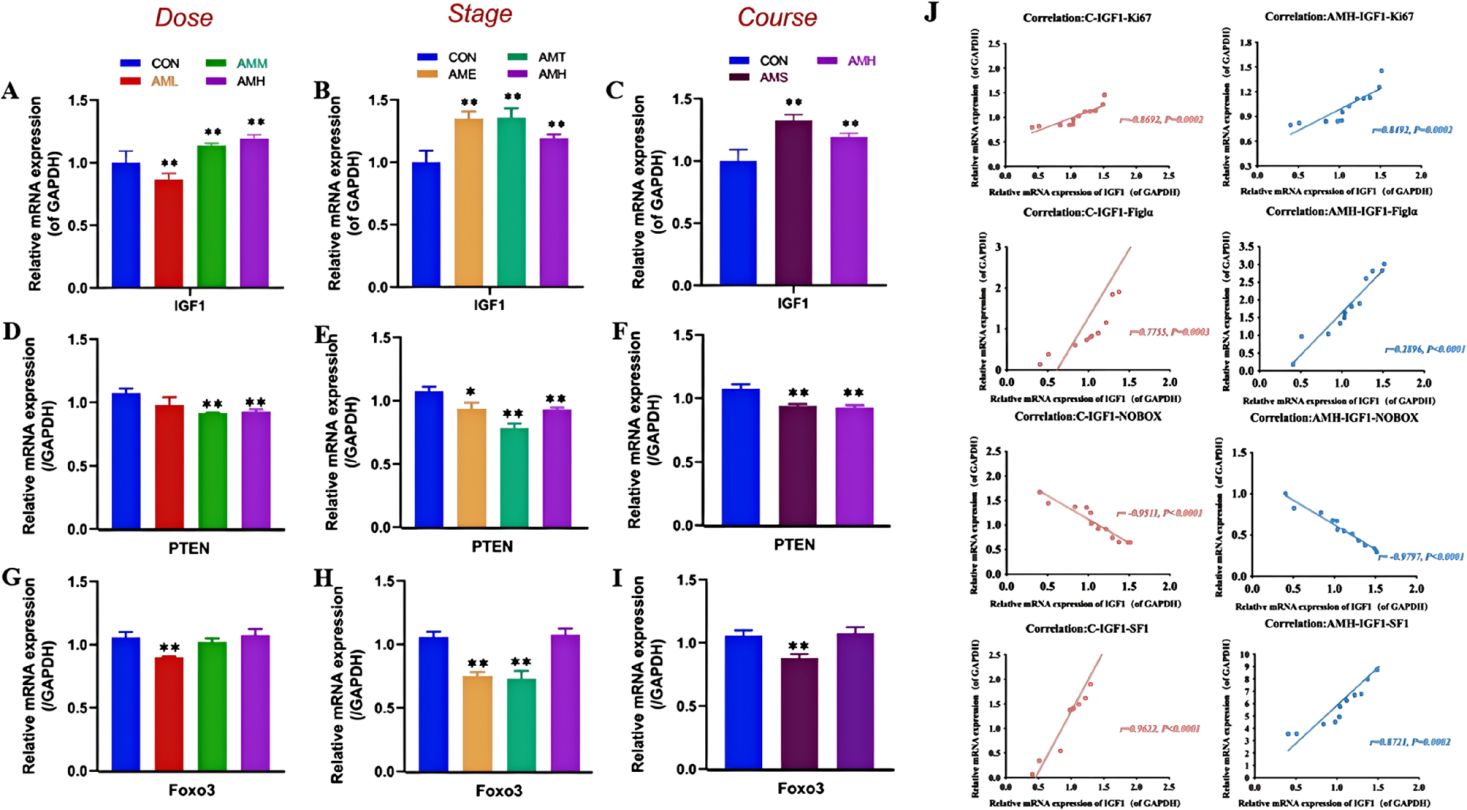
Effects of different doses, stages and courses of prenatal amoxicillin exposure on IGF1 and PTEN-Akt-Foxo3 signaling pathway in fetal ovary. **A**-**I** Relative mRNA level of *Igf1*, *Pten* and *Foxo3* in fetal ovary, *n* = 12 per group; Mean ± S.E.M., ^*^P < 0.05, ^**^P < 0.01 *vs.* CON. (J) Correlation of IGF1 and Ki67, Figlα, NOBOX

To understand how PAmE affects offspring ovary development and function, we studied key markers in the *Igf1* and *Pten-Akt-Foxo3* pathways, crucial for oocyte and pre-granulosa cell development during embryo growth. Results revealed dose-dependent effects: compared to the control group, AML decreased *Igf1* and *Foxo3* mRNA expression (*P* < 0.01, Fig. 6A, G), while AMM and AMH increased *Igf1* but decreased *Pten* mRNA expression (*P* < 0.01, Fig. 6D). Throughout pregnancy stages, PAmE increased *Igf1* but decreased *Pten* mRNA expression (*P* < 0.05, *P* < 0.01, Fig. 6B, E). The mRNA expression of *Foxo3* was suppressed in AME and AMT groups, particularly in AMT group (*P* < 0.01, Fig. 6H). Single and multiple-course of PAmE increased *Igf1* (*P* < 0.01, Fig. 6C) but decreased *Pten* mRNA expression (*P* < 0.01, Fig. 6F), more pronounced in the single-course group. AMS also decreased Foxo3 mRNA expression (*P* < 0.01, Fig. 6I). Notably, *Igf1* correlated positively with *Ki67* and *Nobox* mRNA expression (*P* < 0.05, *P* < 0.01, Fig. 6J). Overall, PAmE alters the mRNA expression of *Igf1* and *PTEN-Akt-Foxo3* pathway in fetal mouse ovaries, notably in high-dose, early to mid-pregnancy, and single-course exposure.

## Discussion

### Construction and rationale of different doses, stages, and courses of PAmE mouse models

The intrauterine period is a critical phase for individual growth and development, easily influenced by external factors (Segal and Giudice 2019). Prenatal medication, a key focus in clinical research, can greatly affect this period (Malm and Ellfolk 2016). Amoxicillin, commonly used during pregnancy to treat infections like chlamydia and E. coli (Adane et al. 2022; Johnson et al. 2021), is typically given at a dose of 1500 mg/day (Johnson et al. 2021; Jacobson et al. 2001; Nishijima et al. 2020). This dose was converted into a mouse dose of 308.5 mg/kg·d according to the body surface area ratio (3:37) (Flint and Hall 2022). In this study, we created three dose groups (AML, AMM, AMH) to simulate clinical use. Since amoxicillin is used at all stages of pregnancy (the first, second and third trimesters) (Nakitanda et al. 2024), and it is known that ovarian development has obvious temporal and spatial effects. In mice, fetal ovaries start developing around GD9.5, progressing to produce estrogen and develop follicles (Barnett et al. 2006). In this study, based on the characteristics of human and mouse fetal ovarian development (Pryor et al. 2000), we established exposure groups in the early (AME), mid (AMT), and late pregnancy (AMH), corresponding to the use of amoxicillin in the first, second, and third trimester of human pregnancy respectively, to observe the effects of exposure at different times during pregnancy on fetal ovarian development. Meanwhile, the treatment course effect is also one of the clinical issues that this study focuses on. Clinically, the course of amoxicillin administration during pregnancy varies from 1 to 8 weeks (Nishijima et al. 2020; Schulz et al. 2022). In this study, we simulated clinical medication and observed the effects of different courses of exposure during pregnancy on fetal ovarian development through single-course (AMS) and multi-course (AMH) administration. Our goal was to investigate how PAmE affects the development of fetal ovaries, both structurally and functionally. It is well documented that in mammals, female fertility is determined by the size of the ovarian oocyte reserve at birth and the rate of depletion (Felici et al. 2005). Our findings indicate that varying doses, stages, and treatment courses of PAmE in fetal rats induce abnormal changes in ovarian structure, including a reduction in oocyte numbers, disruption of germ cell cysts, and premature formation of primordial follicles. These changes were more significant in AML、AMM and AMS groups. This phenomenon can be attributed to the multifaceted impact of maternal drug exposure during pregnancy on the morphological and functional development of the fetus, which involves a “multi-pathway” effect (Lu et al. 2023). Specifically, this combined effect may result in the relationship between drug dosage and biological response not consistently manifesting as a linear curve, thus leading to the atypical nature of the dose-response relationship. However, a single high-dose administration (on GD16 for one day) caused a transient yet significant increase in amoxicillin concentration in the blood of both the mother and the fetus, directly damaging ovarian germ cells *via* the placental barrier. Conversely, in the multi-course group (administration from GD16 to GD18 over three consecutive days) had no significant effect, which may be due to the developmental plasticity of the fetus involving adaptive or compensatory changes.

### PAmE leads to aberrant proliferation and apoptosis in fetal ovaries

The balance of homeostasis between cell proliferation and apoptosis is critical for coordinating the functions of diverse cell populations in complex multicellular organisms. In mice, primordial germ cells (PGCs) undergo rapid proliferation via cell division immediately following their migration to the gonadal ridge at GD9.5. Meiosis is initiated at GD13.5 and remains in meiotic prophase I. From GD18 to birth, a significant number of oocytes undergo programmed cell death via apoptosis, while primordial germ cells continue to proliferate in preparation for primordial follicle formation (Felici et al. 2005). This orderly process of cell proliferation and apoptosis is essential for establishing a defined ovarian morphology and ensuring proper functionality. However, exposure to adverse environment during pregnancy can disrupt this process, affecting the development of fetal ovarian cells (Angenard et al. 2010). It is well established that in female mice, the somatic cell components of the ovaries appear to exert minimal influence on germ cells prior to birth, whereas germ cells significantly impact the somatic cell lineage within the fetal ovaries (McLaren 2000). When PGCs colonize the genital ridge (GD9.5), germ cells undergo rapid proliferation, leading to a significant decrease in the proportion of accompanying somatic cells (McLaren 2000). PCNA is a critical regulatory factor during ovarian follicular development. It is predominantly expressed in oocytes at birth and plays a role in promoting oocyte apoptosis and primordial follicle assembly (Xu et al. 2011). The apoptotic marker gene caspase-3 is essential for promoting granulosa cell apoptosis. In our findings, the expression of PCNA in the fetal ovaries of the AME and AMT groups was lower than that in the control group, whereas it increased in the AMH group. This may be attributed to the fact that the first and second trimesters of pregnancy are critical periods for germ cell proliferation. During this time, exposure to amoxicillin inhibits germ cell proliferation, thereby reducing the number of germ cells present in the ovaries at birth. Consequently, fetal ovaries mitigate oocyte loss by suppressing PCNA expression and delaying the assembly of primordial follicles. These fetal adaptations might be linked to developmental plasticity, wherein exposure to an unfavorable intrauterine environment elicits an “immediate adaptive response” to ensure short-term survival (Hanson and Gluckman 2014). These findings suggest that PAmE promotes somatic cell proliferation and suppresses apoptosis. However, in cases of high-dose and multi-course treatment, exposure during the first and second trimesters of pregnancy inhibits oocyte proliferation and enhances oocyte apoptosis during the third trimester, thereby impacting primordial follicle assembly and ovarian reserve formation.

### PAmE can impair multicellular functions in fetal ovaries

The ovaries consist of different cell types, each with its own role in ovarian function. Granulosa cells, found in the ovarian cortex, mainly produce and release hormones. Studies have shown that adverse conditions during pregnancy can affect the hormone production function of these cells in fetal mouse ovaries (Lv et al. 2021; Lv et al. 2018). SF1 is a key regulator of hormone production, controlling enzymes like StAR and CYP19 (Pelusi et al. 2008). Our research results indicate that, although the levels of fetal blood estrogen in each group of PAmE did not exhibit significant changes, SF1, as well as the steroidogenic enzymes StAR and P450scc, showed substantial increases. These findings suggest that PAmE may initially affect SF1 in the fetal ovary, subsequently influencing estrogen synthesis function. This outcome could also be associated with the promotion of fetal ovarian somatic cell proliferation by PAmE; however, the precise mechanism requires further investigation.

Apart from granulosa cells, germ cells are vital for fetal ovary development. Proper ovarian development during fetal life is crucial for producing healthy oocytes later. Primordial germ cells migrate and undergo changes, eventually becoming oocytes. Various genes and pathways are involved in germ cell development, ensuring normal ovarian growth. Oocytes regulate follicle formation and maturation by expressing specific factors, such as Nobox and BMP15 (Wang et al. 2020). Nobox can regulate the expression of BMP15 (Rajkovic et al. 2004). Mice lacking the Nobox gene exhibit accelerated oocyte loss after birth and fail to undergo the transition from primordial follicles to growing follicles (Rajkovic et al. 2004). These genes also play a critical role in human follicular development (Suzumori et al. 2007). For instance, the development of premature ovarian failure (POF) may be associated with the loss of function of the Nobox gene (Franca et al. 2017; Sassi et al. 2021). Figlα plays a critical role in regulating the rupture of oocyte nests or follicular assembly, whereas Nobox is more involved in modulating post-formation follicular development (Pangas and Rajkovic 2006; Zheng and Dean 2007). In this study, the expression Figlα was upregulated in each group of PAmE, whereas the expression of Nobox was downregulated. These findings suggest that PAmE may promote the rupture of fetal ovarian oocyte cysts, induce oocyte apoptosis, facilitate primordial follicle assembly, and potentially accelerate the activation and depletion of primordial follicles postnatally, thereby shortening the reproductive lifespan of females.

### The involvement of IGF1 and the PTEN/AKT/Foxo3a signaling pathway in PAmE-induced ovarian damage

Phosphatase and tensin homolog (PTEN) is a negative PI3K/AKT signaling pathway regulator and has been considered a universal tumor suppressor gene since 1997. However, defects in PTEN in primordial and primary follicle oocytes suggest that the PTEN/Akt/FOXO3 signaling pathway in oocytes is crucial for maintaining the primordial follicle pool. The regulation of the PTEN/Akt/FOXO3 signaling pathway can accelerate or decelerate the rate of ovarian reserve depletion, leading to conditions such as POF or an extended reproductive period. Studies have shown that mice lacking PTEN in oocytes (homozygous phosphatase-tensin homolog on chromosome ten, PTEN-loss mice) activate the entire pool of primordial follicles. Subsequently, all primordial follicles are exhausted in early adulthood, resulting in POF (Reddy et al. 2008). Foxo3a, a transcription factor and a substrate of Akt, has been shown to inhibit follicle activation (Castrillon et al. 2003). IGF1 is a positive regulator of the PI3K/AKT signaling pathway and acts on granulosa cells and oocytes in the ovary through both paracrine and autocrine modes, regulating cell proliferation, differentiation, survival, steroid synthesis, and oocyte maturation (Mazerbourg et al. 2003; Bezerra et al. 2018). Our previous animal experiments confirmed that exposure to exogenous substances during pregnancy, such as caffeine, dexamethasone, and nicotine, can inhibit ovarian development by reducing IGF1 signaling and suppressing SF1 expression in offspring ovaries (Lv et al. 2018, 2021; Fan et al. 2019). In our study, we found that PAmE increased IGF1 and reduced Pten and Foxo3a expression in fetal ovaries, promoting cell growth and SF1 expression. It is suggested that the IGF1/Pten/AKT/Foxo3a signaling pathway may be involved in the enhanced steroid synthesis function of fetal ovarian pregranulosa cells and the inhibition of primorprimitive follicular assembly caused by PAmE. In conclusion, PAmE may enhance the steroid synthesis function of pre-granulosa cells in the fetal ovary and promote the assembly of primordial follicles. By upregulating IGF1 expression in the fetal ovary and inhibiting the Pten/AKT/Foxo3a signaling pathway, it could potentially reduce ovarian reserve. Additionally, PAmE may lead to excessive activation of primordial follicles postnatally, thereby disrupting the balance between the activation and quiescent states of primordial follicles and causing premature depletion of the primordial follicle pool.

The intrauterine development of ovarian dysplasia may involve several mechanisms, including increased sister chromatid exchange frequency and chromosomal aberrations, disrupted signaling pathways associated with follicular development, an imbalance between oxidative defense and oxidative stress, abnormal epigenetic modifications of developmental genes, and alterations in intrauterine developmental programming (Huang et al. 2022; National Toxicology 1988). The primary objective of this study was to confirm that different stages, doses, and courses of amoxicillin exposure during pregnancy exert distinct effects on the ovarian development of female offspring. The evaluation criteria predominantly focused on morphological and functional aspects of ovarian development. However, the underlying mechanisms and long-term consequences remain ambiguous and unverified, which will be a critical focus of our future research endeavors.

## Conclusion

In this study, we created mouse models with different doses, stages and courses of PAmE to mimic how amoxicillin is used clinically. We observed that the ovarian development of PAmE offspring was compromised, primarily characterized by enhanced somatic cell proliferation, suppressed apoptosis, increased oocyte apoptosis, elevated estrogen synthesis and premature follicular assembly. The most significant changes to fetal ovarian pre-granulosa cells occurred with late-pregnancy, multiple-courses, and clinical dose groups, while the damage of fetal oocytes was the most severe in the early to mid-pregnancy, single-course and clinical dose groups, possibly influenced by the IGF1/Pten-AKT-Foxo3a pathway. Given amoxicillin’s widespread use in pregnant women with infections, it’s crucial to reassess its impact on human reproductive health with better evidence. This study lays the groundwork for guiding amoxicillin use during pregnancy and understanding its effects on ovarian development, adult disease risks, and prenatal programming.

## Data Availability

No datasets were generated or analysed during the current study.
